# Evaluation of Ocular Higher-Order Aberrations in Patients with Migraine: A Comparative Study

**DOI:** 10.22336/rjo.2026.31

**Published:** 2026

**Authors:** Ashutosh Kumar Mishra, Ruchi Shukla, Pragati Garg, Aparajita Shukla, Mukesh Shukla, Nilakshi Banerjee

**Affiliations:** 1 Department of Neurology, All India Institute of Medical Sciences, Raebareli, Uttar Pradesh, India; 2 Department of Ophthalmology, All India Institute of Medical Sciences, Raebareli, Uttar Pradesh, India; 3 Department of Community Medicine, All India Institute of Medical Sciences, Raebareli, Uttar Pradesh, India

**Keywords:** migraine, higher-order aberrations, aberrometry, visual aura, photophobia, coma aberration, HOA = Higher order aberrations, SD-OCT = Spectral Domain Optical Coherence Tomography, DED = Dry Eye Disease

## Abstract

**Objective:**

To assess and compare higher-order aberrations (HOAs) in migraine patients versus healthy controls, and to explore potential correlations between aberrometric parameters and migraine characteristics.

**Methods:**

A cross-sectional study was conducted involving 99 migraine patients (32 episodic and 67 chronic) and 99 age- and sex-matched controls. Comprehensive ophthalmologic examinations and wavefront aberrometry (using Huvitz HRK-8000A Autorefractor/Keratometer based on a Hartmann–Shack principle) were performed on all participants. HOAs, including spherical aberration, coma, trefoil, and quadrafoil, were quantified. Statistical analyses examined differences between groups and correlations with clinical migraine data.

**Results:**

Migraine patients exhibited higher total HOAs, but the result was not statistically significant compared to controls. Coma and oblique trefoil aberrations were notably elevated in the migraine patients. Oblique trefoil was significantly higher in cases than in controls. However, no correlation was found between HOA magnitude and migraine disease duration. Statistically significant correlation was found when comparing episodic versus chronic migraine cases.

**Discussion:**

Increased higher-order aberrations, especially oblique trefoil, are linked to chronic migraine, indicating that even with normal acuity, small optical variables may cause visual disruptions. Although more long-term research is required to establish causality, these results point to a combination of ocular and neurological mechanisms.

**Conclusions:**

Migraine patients demonstrate increased higher-order ocular aberrations, which may contribute to their visual symptoms. Aberrometry may serve as a valuable tool in identifying visual triggers and customizing optical interventions for migraine management.

## Introduction

Migraine is a prevalent and often incapacitating neurovascular disorder, affecting approximately 15-20% of the global population, with a significantly higher burden observed among women, particularly during their reproductive years [1]. Migraine attacks typically involve a unilateral throbbing headache, accompanied by nausea, aura, multisensory hypersensitivity (to light, sound, smell), and marked fatigue, with symptoms varying across prodrome, aura, headache, and postdrome phases [2]. Migraines are the second most common cause of disability globally among people under 60, according to the Global Burden of Disease Study 2024 [3].

Despite its widespread impact, the underlying pathophysiology of migraine remains complex and multifactorial. Key mechanisms include cortical spreading depression, which underlies aura; trigeminovascular activation, which causes neurogenic inflammation and pain; and sensory hypersensitivity due to central sensitization. Additionally, hypothalamic dysfunction, metabolic imbalance, and oxidative stress are increasingly recognized as important contributors across different migraine phases [4].

The link between migraine and the eye is both complex and clinically important. Migraines can affect various eye structures from the eyelids and pupils to the optic nerve and retina, often presenting as visual aura or eye pain. Many undiagnosed patients first seek eye care for symptoms such as photophobia, which can mimic other eye conditions [5].

Recent research has shifted attention toward the eye’s optical system as a potential contributor to visual discomfort and photophobia in migraine. The tear film plays a crucial role in ocular optics as it forms the eye’s primary refractive surface. Irregularities or instability in the tear film can lead to optical distortions, with several studies showing that such fluctuations contribute to increased irregular astigmatism and aberrations [6].

When the eyes stay open for extended periods, the tear film begins to break up, leading to surface irregularities and an increase in HOAs. Dry eye, characterized by inadequate tear production or instability, disrupts the smoothness of the ocular surface. Numerous studies have shown that this disruption leads to elevated HOAs relative to healthy eyes, ultimately impairing visual quality and reducing optical performance [7,8].

HOAs are strongly associated with migraine in a few studies. Third-order, coma, spherical, and fifth-order aberrations were found to be significantly higher in migraine patients than in controls. This implies that optical HOAs might contribute to the pathophysiology of migraines, perhaps by impairing retinal image quality and inciting attacks. Further connecting ocular surface health abnormalities and migraine development are dry eye-induced HOAs, particularly coma and trefoil, which are important migraine triggers. Thus, aberrometers could be used as screening and diagnostic tools to identify people at risk for migraines [9].

Confounding variables such as age, refractive error, ocular surface conditions, and measurement variability can alter results, and findings in the literature are not always consistent. Thus, further research is warranted to clarify the relationship between ocular aberrations and migraine. The present study aims to evaluate and compare wavefront aberrations in migraine patients and healthy controls using an aberrometer. By identifying any consistent patterns, this research seeks to enhance our understanding of migraine pathophysiology and explore the potential role of ocular aberrations in its diagnosis.

## Materials and methods

In this study, we recruited 99 migraine patients and 99 age- and gender-matched controls between July 2023 and March 2025 from the outpatient Departments of Neurology and Ophthalmology at a tertiary care center in India.

Participants in our study were patients who met the International Classification of Headache Disorders-III (ICHD-III) diagnostic criteria for episodic or chronic migraine [10]. These migraine patients were in the age group of 18-50 years. Excluded from the study were patients with secondary headaches, structural brain injuries, central nervous system disorders, ocular diseases such as glaucoma, dry eye disease, ocular surface disorders, cataract, optic disc edema, or retinal pathology, and pregnant women.

The control group consisted of 99 gender and age-matched individuals who had never experienced a migraine attack and had no prior history of neurological or ocular disorders.

A comprehensive neurological examination and a semi-structured interview with a neurologist were conducted for each patient to assess their headaches thoroughly. The demographic and clinical parameters of the patients were recorded, including age, gender, and migraine-related variables such as aura, migraine episode duration, headache attack frequency per month, and duration of migraine disease.

A comprehensive ophthalmic examination was performed, including best-corrected visual acuity using Snellen’s chart, measurement of central corneal thickness, intraocular pressure using Goldmann applanation tonometry, slit lamp biomicroscopy, Retinal Nerve fiber Layer thickness assessment with the help of SD-OCT (Cirrus 5000, ZEISS Medical Technology, Oberkochen, Germany), and aberrometric analysis using a wavefront aberrometer (Huvitz HRK-8000A Autoref-keratometer, Korea). Measurements included third-order aberrations (e.g., coma, trefoil) and fourth-order aberrations (e.g., spherical aberration, secondary astigmatism). The root-mean-square (RMS) values for each aberration type were recorded for statistical analysis.

Microsoft Excel (Microsoft Corporation, Redmond, WA) was used to examine the data. For migraine patients and controls, the normally distributed data were presented as mean and standard deviation (SD). In contrast, the non-normally distributed data were presented as median and interquartile range (IQR). In regularly distributed quantitative data, the comparison between migraine patients and control groups was performed using the independent sample t-test; in non-normally distributed quantitative data, the comparison was made using the Mann-Whitney test to assess the association between HOAs and clinical migraine parameters (e.g., duration of disease), and Spearman’s rank correlation coefficient (r) was calculated. Subgroup analysis was also conducted to compare aberration values between patients with episodic and chronic migraine, and the corresponding p-values were reported.

A p-value < 0.05 was considered statistically significant for all analyses. The results were interpreted in the context of the clinical relevance of HOA variations in migraine pathophysiology.

## Results

In this study, 99 migraine patients and 99 controls were included. There was no statistically significant difference between the two groups (p = 0.41, 0.44, respectively) with respect to age and gender. Table 1 illustrates the demographic characteristics of migraine patients and controls.

**Table 1:** Comparison between two groups based on age and gender

Variable	Cases (n=99)	Control (n=99)	^#^p-value
Age in years Mean ± SD [Median; IQR]	30.17 ± 8.52 30.00; 22.00-38.00	30.89 ± 5.63 32.00; 30.00-34.00	0.41
^$^Gender			
Male	32	34	0.44
Female	67	65	

# Mann Whitney U test; ^$^Chi-square test *p<0.05 considered as significant

There was no statistically significant difference in the mean total HOAs between migraine patients and controls (p = 0.42; Table 2).

**Table 2:** Comparison between two groups concerning the total higher order aberration

Variable	Cases (n=99)	Control (n=99)	^#^p-value
Total high order Mean ± SD [Median; IQR]	0.124 ± .165 0.107; 0.079-0.142	0.122 ± .100 0.101; 0.078-0.132	0.42

# Mann Whitney U test; *p<0.05 considered as significant

**Fig. 1:**
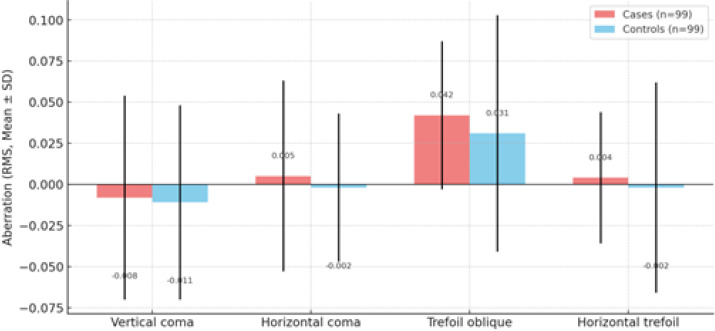
Comparison of third-order aberrations between migraine patients and healthy controls. Bars represent mean RMS values with error bars showing standard deviation (SD)

The correlations between the third-order aberrations of migraine patients and healthy controls are shown in Table 3. Statistically significant correlations were found for oblique trefoil, a third-order aberration, which was significantly higher in migraine patients than in controls (*p* = 0.02; Table 3; Fig. 1), indicating subtle but measurable optical distortion. Other parameters, vertical coma, horizontal coma, and horizontal trefoil, did not show a significant difference between the two groups.

**Table 3:** Comparison between the two groups concerning third-order aberrations

Aberration Mean ± SD [Median; IQR]	Cases (n=99)	Control (n=99)	^#^p-value
RMS Vertical coma	-0.008 ± .062 0.000; -0.045 - 0.029	-0.011 ± .059 -0.013; -0.044 - 0.021	0.26
RMS horizontal coma	0.005 ± .058 0.006; -0.023 - 0.025	-0.002 ± .045 0.001; -0.023 - 0.029	0.70
RMS Trefoil oblique	0.042 ± .045 0.039; 0.010 - 0.075	0.031 ± .072 0.025; 0.004 - 0.056	*0.02
RMS horizontal trefoil	0.004 ± .040 0.001; -0.024 - 0.033	-0.002 ± .064 0.002; -0.015 - 0.023	0.70

# Mann Whitney U test; *p<0.05 considered as significant

No significant differences were observed in fourth-order aberrations between the two groups, including spherical aberration, quadrafoil components, and secondary astigmatism (*p > 0.05*; Table 4).

**Table 4:** Comparison between the two groups concerning fourth-order aberrations

Aberration Mean ± SD [Median; IQR]	Cases (n=99)	Control (n=99)	^#^p-value
RMS Oblique Quadrafoil	0.010 ± .104 -0.001; -0.016 - 0.012	0.002 ± .030 0.002; -0.014 - 0.013	0.57
RMS horizontal Quadrafoil	0.020 ± .075 0.015; -0.005 - 0.029	0.008 ± .034 0.005; -0.010 - 0.024	0.10
RMS spherical Quadrafoil	-0.012 ± .029 -0.018; -0.032 - 0.001	-0.015 ± .028 -0.019; -0.036 - 0.003	0.53
RMS secondary astigmatism	0.006 ± .040 0.004; -0.013 - 0.021	0.004 ± .024 0.006; -0.012 - 0.016	0.92
RMS oblique secondary astigmatism	0.008 ± .064 0.002; -0.006 - 0.011	0.004 ± .029 0.005; -0.006 - 0.012	0.51

# Mann Whitney U test; *p<0.05 considered as significant

There was no significant correlation between the duration of migraine and total HOAs (r = -0.053, p = 0.59; Table 5), indicating that longer disease duration did not necessarily result in greater optical distortion.

**Table 5:** Correlation of high-order aberration in migraine cases with duration of disease

Aberration	^#^Correlation coefficient	p-value
High-order aberration	-0.053	0.59

# Spearman correlation coefficient

**Fig. 2:**
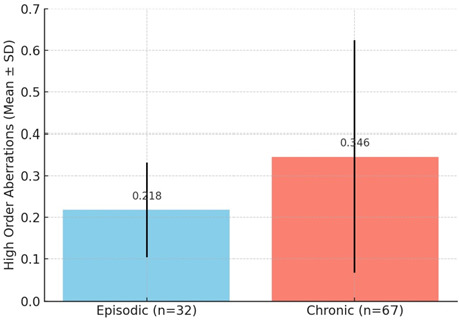
Comparison of higher-order aberrations (HOAs) between episodic and chronic migraine patients. Bars represent mean HOA values with error bars indicating standard deviation (SD)

A subgroup analysis revealed that chronic migraine patients had significantly higher HOAs compared to episodic migraine patients (p = 0.02, Table 6; Fig. 2). This finding suggested that prolonged or more frequent migraine episodes might be associated with greater ocular aberrations.

**Table 6:** High-order aberrations with the type of migraine

Aberration Mean ± SD [Median; IQR]	Type of migraine		p-value
	Episodic (n=32)	Chronic (n=67)	
High-order aberrations	0.218 ± 0.114 0.203; 0.123-0.290	0.346 ± 00.279 0.274; 0.155-0.411	0.02

## Discussion

Using a wavefront aberrometer, this case-control study examined the presence and features of ocular higher-order aberrations (HOAs) in migraineurs by contrasting them with age- and sex-matched healthy controls. According to our research, migraine patients with chronic migraine have higher total HOA root mean square values than those with episodic migraine, and they also exhibit a marked increase in oblique trefoil, a third-order aberration. These findings imply a connection between the pathophysiology of migraines and minute optical flaws, which could contribute to the visual abnormalities frequently reported by migraineurs.

Recurrent, usually unilateral, pounding headache episodes, nausea, increased sensitivity to light, sound, or smells, and occasionally sensory disturbances known as aura are the hallmarks of migraine, a complex neurological condition. These episodes occur in distinct stages, beginning with a premonitory stage in which people may experience modest warning signals such as mood changes or neck stiffness. Aura is most commonly associated with visual phenomena such as flickering lights, blind spots, or shimmering shapes that persist for many minutes. After the pain has subsided, many people have a postdrome phase characterized by fatigue, mental fog, or increased sensitivity, which is commonly referred to as “migraine hangover”. Migraines can be episodic or chronic, depending on how many times they occur per month. Because the eyes are strongly involved in how we process sensory information, migraines, particularly those with visual aura, can have a major impact on visual comfort and quality of life [11-13]. These symptoms may not always be explained by overt ocular pathology, suggesting that subclinical abnormalities in optical and cortical processing may be involved.

HOAs are minute flaws in the eye’s optical system that reduce the quality of retinal images. Since HOAs cannot be corrected with most contact lenses or ordinary glasses, they are more challenging to treat than common vision abnormalities like myopia or astigmatism [14,15].

Ocular wavefront distortions can be measured objectively and non-invasively using an aberrometer, which gives information about how light enters the eye and how it may be distorted before it reaches the retina [16].

Even though standard visual acuity is unaffected in many migraineurs, it is an insensitive predictor of the visual impact of HOAs. Instead, contrast sensitivity is a more accurate measure of the functional impacts of these abnormalities.

Even when the sampling density of retinal photoreceptors limits spatial resolution, correcting HOAs can increase retinal image contrast at low spatial frequencies, thereby greatly improving perceived sharpness and clarity of vision [17]. This is especially true for migraineurs, who frequently report symptoms like glare, halos, and visual fatigue even though their acuity is normal. This suggests that the problem is with visual quality rather than acuity. Such contrast reductions brought on by aberration may worsen cortical hyperexcitability and make migraine attacks more likely, especially in difficult-to-see environments.

Tear film instability, which is frequently observed in dry eye disease (DED), is a significant yet often overlooked factor that increases HOAs. Uneven optical surfaces and a discernible rise in HOAs, especially after blinking, are caused by localized tear film break-up, which is caused by disruptions in the tear film, which forms the eye’s fundamental refractive interface. Over time, it has been demonstrated that this tear film irregularity directly lowers optical quality and raises the frequency and unpredictability of HOAs [18,19].

There is mounting evidence of a close connection between dry eye disease and migraines. According to several studies, DED is more common in migraineurs, especially those who have aura or have had the condition for a longer period of time [20,21]. Both conditions share a common pathway via the trigeminal nerve, which innervates the cornea and ocular surface and is also central to migraine pathogenesis [22]. Trigeminal hyperexcitability may explain the overlapping symptoms of ocular discomfort, photophobia, and visual sensitivity frequently observed in both disorders [23].

Thus, increases in HOAs associated with dry eye could place additional strain on the visual system in migraineurs. Particularly in situations involving photic stress or visually demanding environments, the presence of even low-grade HOAs may work in concert with a sensitized trigeminovascular system to lower the threshold for migraine activation.

Although overall HOA and RMS values did not differ significantly between cases and controls in our study, a significantly higher oblique trefoil in migraineurs indicated asymmetrical distortions that might contribute to visual discomfort or instability in image perception during daily activities.

The clinical importance of such third-order aberrations is underscored by studies showing their correlation with glare, halos, and difficulty with night driving even in eyes with 6/6 visual acuity [16]. It is likely that even slight optical flaws, such as trefoil, could worsen visual discomfort and possibly trigger headaches in migraineurs, who already exhibit cortical hyperresponsiveness to visual stimuli. Furthermore, the higher HOA values observed in chronic migraine may be the result of repeated trigeminovascular activation’s cumulative effects, or they may be the visual system’s adaptive reactions to ongoing cortical stress.

From a neurological standpoint, migraine has been linked to changes in the retina and visual cortex. Research employing functional magnetic resonance imaging and visual evoked potentials has shown that during interictal phases, cortical excitability increases and connectivity in visual areas changes [24]. At the same time, ocular imaging studies have observed slight thinning of the ganglion cell complex and the retinal nerve fiber layer (RNFL), suggesting that structural alterations may accompany functional disruptions [25,26]. The idea that migraine is not only a disorder of the central nervous system but may also involve ocular and optical components is further supported by our findings, which add another dimension to this evidence by identifying differences in wavefront aberrations.

However, the lack of a statistically significant relationship between the duration of the disease and total HOA raises the possibility that these optical aberrations are a natural feature of migraineurs rather than a progressive phenomenon, supporting the idea that some people have a pre-existing susceptibility that may be related to connective tissue properties, ocular geometry, or neurovascular feedback mechanisms that predispose them to both migraine and optical irregularities.

The study had certain limitations that warrant consideration. First, its cross-sectional design restricted the ability to establish causal relationships between migraine and HOAs. Longitudinal studies are required to determine whether HOAs precede migraine onset or develop because of chronic disease. Second, although we excluded participants with known ocular surface disease, subtle or subclinical tear film instability could still have influenced the measurements, given its well-documented impact on HOAs. Third, we relied on a single aberrometer system (Huvitz HRK-8000A), and inter-device variability may limit the generalizability of findings. Fourth, migraine severity, frequency, and medication history were self-reported, potentially introducing recall bias. Additionally, the relatively modest sample size, particularly in the subgroup analysis of episodic versus chronic migraine, might have limited the statistical power to detect smaller aberrometric differences.

Future research should focus on multicentre, longitudinal studies with larger sample sizes to validate these findings across diverse populations. Incorporating simultaneous assessment of ocular surface parameters, tear film stability, and corneal biomechanics could provide a more comprehensive understanding of the interplay between migraine and ocular optics. Functional vision tests, such as contrast sensitivity and glare disability assessments, might help bridge the gap between objective aberrometric findings and subjective patient symptoms. Moreover, integrating neuroimaging with ocular wavefront analysis could uncover mechanistic links among cortical hyperexcitability, trigeminal pathways, and ocular optical disturbances. Ultimately, exploring customized optical correction strategies, such as wavefront-guided spectacles or contact lenses, might open new avenues to reduce visual triggers and improve the quality of life for migraine patients.

## Conclusion

This study demonstrated a significant association between migraine, particularly chronic migraine, and increased ocular higher-order aberrations, with oblique trefoil emerging as a notable contributor. Even in the absence of decreased visual acuity, these minute optical abnormalities might be the cause or aggravating factor of the visual discomfort commonly experienced by migraineurs. Even though more longitudinal research is needed, our results showed that wavefront aberrometers could be a useful adjunct in the clinical evaluation of migraine, offering insights into the neuro-ophthalmic aspects of the condition and creating opportunities for tailored optical correction techniques to enhance the visual comfort and quality of life of those affected.
